# Impact of COVID-19 Pandemic on Cardiovascular Healthcare in Croatia: A Comprehensive Nationwide Survey

**DOI:** 10.3390/diseases12030042

**Published:** 2024-02-22

**Authors:** Josip Lukenda, Josip Andelo Borovac

**Affiliations:** 1 Department of Internal Medicine, University Hospital “Sveti Duh”, 10000 Zagreb, Croatia; lukendaj@hotmail.com; 2 Cardiovascular Diseases Department, University Hospital of Split, 21000 Split, Croatia; 3 Department of Pathophysiology, University of Split School of Medicine, 21000 Split, Croatia; 4 University Department of Health Studies, University of Split, 21000 Split, Croatia

**Keywords:** cardiology services, Croatia, COVID-19, pandemic, healthcare, national sample, interventional cardiology, hospitals, admissions, outpatient

## Abstract

The impact of the COVID-19 pandemic on cardiovascular healthcare in Croatia remains largely unexplored. This study aimed to compare the utilization and provision of cardiovascular services during the pre-pandemic (2017–2019) and pandemic (2020–2021) periods, leveraging nationwide data from the Croatian Health Insurance Fund, which covers 95% of all insurance claims in the country. Our findings reveal that while the use of coronary angiographies decreased during the pandemic, there was a notable increase in the utilization of advanced heart failure treatment modalities and percutaneous coronary interventions, particularly in the context of acute myocardial infarction. Additionally, transcatheter aortic valve implantations saw a significant rise during the pandemic period. Furthermore, laboratory diagnostic testing for troponin and natriuretic peptides experienced a marked increase, while the utilization of most other cardiovascular services remained stable or showed only minor declines compared to the pre-pandemic era. These observations suggest that the Croatian cardiovascular healthcare system displayed resilience during the COVID-19 pandemic, successfully maintaining and even expanding access to various diagnostic and interventional procedures despite facing widespread societal and logistical challenges.

## 1. Introduction

The coronavirus disease (COVID-19) pandemic was declared by the World Health Organization (WHO) on 11 March 2020. This was preceded by a forewarning from the same institution on 30 January 2020 [1]. From 571 cases of COVID-19 observed on 22 January 2020, in China, only one month later, on 16 February, the number of COVID-19 cases reached 51,857 cases globally in 25 countries [2]. Overall, we can realize that the coronavirus pandemic began at the beginning of 2020.

As the number of COVID-19 cases increased, a number of epidemiological interventions were introduced worldwide, and patients’ concerns about and fear of visiting hospitals increased. This resulted in a sharp decrease in the number of non-COVID hospital appointments, admissions, and medical procedures. An analysis of approximately one million medical admissions from 201 hospitals in 36 states across the USA revealed a substantial decline in non-COVID-19 admissions from February to April 2020, exceeding 20% but later rebounding to 16% below the pre-pandemic level [3]. In the multicenter European observational registry from 15 centers in 12 countries, 54,331 patients were analyzed, and acute admissions to emergency departments in 2020 decreased by an incidence rate ratio (IRR) of 0.66 compared to 2019 [4]. The influence of the COVID-19 epidemic on non-COVID-19 cases was also recorded in the Republic of Croatia. An analysis of the hospital admission rate in Croatia showed a 21% decrease in the total number of admissions across Croatian hospitals during 2020. A decrease in hospital admissions was observed in some non-elective Diagnosis-Related Groups (DRG classes), such as cancer, stroke, major chest procedures, heart failure, and renal failure. In the same study, the decrease in the number of CVD admissions in Croatia was 26% [5].

As the prevalence of cardiovascular diseases (CVDs) is high worldwide, for example, in the USA, the overall prevalence in adults ≥20 years of age is 49.2% and increases with age in both males and females [6], we can expect there to be a significant impact of the COVID-19 pandemic on CVD prevention, diagnostics, and treatment. Cardiovascular diseases are also the main causes of death in Croatia, and ischemic heart disease represents one-fifth of all deaths [7]. It is expected that the delay in cardiovascular healthcare delivery during the COVID-19 pandemic would negatively affect the cardiovascular outcomes of non-COVID cardiovascular patients in the future.

So far, the impact of the COVID-19 pandemic on cardiovascular healthcare delivery in Croatia has not been comprehensively analyzed at the national level. Therefore, the aim of the present study was to analyze the impact of the COVID-19 pandemic on hospital admissions and determine the magnitude of change in related cardiology services and procedures in Croatia by comparing references from the pre-pandemic (2017–2019) and pandemic (2019–2021) period.

## 2. Materials and Methods

Data for this study were publicly available upon request from the Croatian Health Insurance Fund (CHIF). According to the 2021 census, Croatia has approximately 3.8 million citizens, and the CHIF covers health services for over 95% of the population. The World Bank classifies Croatia as a high-income country (defined as having a gross national income per capita of USD 13,589 or more in 2022). Data from all Croatian non-specialized acute hospitals (including 11 tertiary- and 22 secondary-level hospitals) were included, representing 96% of the country’s inpatient activity.

The CHIF dataset consisted of inpatient data categorized using Australian Refined Diagnosis Related Groups (AR-DRGs), a system based on the Australian DRG system. It employs a combination of ICD-10AM and ICD-10 classifications for diagnoses and Australian Classifications of Health Interventions (ACHI) for procedures. The DRG grouping algorithm used version 5.2, which assigns cases to 671 DRG classes.

Data were collected for the period from 1 January 2017 to 31 December 2021. The pre-pandemic period was defined as 1 January 2017 to 31 December 2019, and the mean values for those three years were calculated. The pandemic period was defined as 1 January 2020 to 31 December 2021, and the mean values for those two years were calculated. To compare the incidence rates of events occurring in these two periods, the incidence rate ratio (IRR) was used as a relative difference measure.

The primary statistical analysis employed an “Odds Ratio” calculator. The Odds Ratio (OR), its standard error, and 95% confidence interval were calculated according to Altman, 1991. A standard normal deviation (z-value) was calculated through formula ln(OR)/SE{ln(OR)}, and the *p*-value represented the area of the normal distribution falling outside ±z. The Odds Ratio calculator can be accessed at https://www.medcalc.org/calc/odds_ratio.php (accessed on 15 November 2023). The *p*-values < 0.05 were considered as statistically significant at all instances.

## 3. Results

As shown in Table 1, hospital admissions for heart failure (HF) patients in Croatia significantly decreased during the pandemic period, dropping from 6021 per year pre-pandemic to 5474 cases per year. Advanced therapeutic methods for HF, including established procedures like heart transplantation, cardiac resynchronization therapy (CRT), and ventricular assist device (VAD) implantations, remained largely unaffected by the pandemic, with only non-significant increases observed. However, extracorporeal membrane oxygenation (ECMO) procedures saw a significant rise during the pandemic period.

Hospital admissions for acute coronary syndrome (ACS) patients in Croatia significantly decreased during the pandemic period, dropping from 11,481 per year pre-pandemic to 10,275 per year, as shown in Table 2. Looking at different types of ACS, admissions for unstable angina (UA) saw a decline, falling from 1910 per year pre-pandemic to 1525 per year during the pandemic. On the other hand, the number of admitted non-ST-segment-elevation myocardial infarction (NSTEMI) patients remained similar while admissions for ST-segment-elevation myocardial infarction (STEMI) decreased from 4311 per year in the pre-pandemic period to 3658 per year during the pandemic period. The largest decrease in admissions was observed for chronic coronary syndrome (CCS), decreasing from 6270 per year in the pre-pandemic period to 4122 per year during the pandemic period.

As Table 3 shows, the total number of coronary angiographies (CAG) in Croatia significantly decreased during the pandemic period, decreasing from 25,938 per year to 20,134 per year. Conversely, the total number of percutaneous coronary interventions (PCIs) remained similar in both periods. On the other hand, the mean number of PCI procedures performed in the setting of acute myocardial infarction (AMI) increased significantly—from 4214 per year in the pre-pandemic period to 4424 per year in the pandemic period.

The number of coronary angiographies performed in AMI patients but without subsequent PCI saw a decrease during the pandemic period. The volume decrease in the number of PCI procedures in the CCS setting was also significant, but less extensive than the drop in the number of admitted CCS patients.

The total number of coronary artery bypass grafting (CABG) cases decreased from 1113 per year in the pre-pandemic period to 894 per year during the pandemic period, but data about preoperative conditions of included patients were not available in the current dataset.

Furthermore, utilization of advanced interventional procedures, such as transcatheter closure of atrial septal defect (ASD), percutaneous transluminal septal myocardial ablation (PTSMA), or percutaneous myocardial biopsy remained similar during both examined periods. In contrast, the number of transcatheter aortic valve implantation (TAVI) procedures doubled from the pre-pandemic to pandemic period.

As shown in Table 4, in-hospital imaging diagnostic procedures such as transesophageal echocardiography (TEE) did not decrease during the pandemic period. Instead, it significantly increased among hospitalized patients, from 3981 cases/year in the pre-pandemic period to 4357 cases/year in the pandemic period. On the other hand, the utilization of in-hospital transthoracic echocardiography (TTE) decreased significantly in the pandemic versus pre-pandemic period. Cardiac magnetic resonance imaging (MRI) performed for admitted patients significantly increased from 283 cases/year in the pre-pandemic period to 369 cases/year in the pandemic period while coronary CT angiography (CCTA) and calcium scoring utilization significantly decreased from 374 cases/year in the pre-pandemic period to 318 cases/year in the pandemic period.

The use of standard laboratory tests for risk stratification among patients with suspected coronary artery disease such as lipid profile decreased significantly during the pandemic period. In contrast to this, the use of troponin testing, a standard laboratory diagnostic test for the work-up of acute chest pain, performed across all Croatian hospitals, increased significantly during the pandemic versus pre-pandemic period. The use of the gold standard laboratory test for the diagnosis of heart failure, natriuretic peptide testing, more than doubled during the pandemic vs. pre-pandemic period.

During the pandemic period, hospital admissions of patients with second/third degree atrioventricular block remained similar compared to the pre-pandemic period as shown in Table 5.

Hospital admissions for sick sinus syndrome, however, decreased significantly, falling from 448 per year in the pre-pandemic period to 356 per year in the pandemic period. Consequently, pacemaker implantations (VVI and DDD modes) also saw a significant decrease, from 3467 per year pre-pandemic to 2936 per year during the pandemic. In contrast, implantable cardioverter defibrillator (ICD) implantations increased from 608 per year in the pre-pandemic period versus 699 per year during the pandemic period.

## 4. Discussion

The COVID-19 pandemic has had a profound impact on the healthcare system, particularly on non-COVID-19 hospital admissions, including both acute and elective cases, as well as the volume of procedures for admitted patients. Notably, cardiovascular diseases, a globally recognized leading cause of morbidity and mortality, were disproportionately affected. A systematic review by Samuel Seidu et al. revealed a substantial decline in admissions for various cardiovascular diseases worldwide, particularly for myocardial infarction (MI), acute coronary syndrome (ACS), and stroke, with reductions reaching up to 73% [8]. However, it is important to note that most studies in this review were published in 2020, with only a minority in 2021 (8.7%).

A comprehensive Croatian study conducted at the end of 2020 underscored a 21% decrease in the total number of hospital admissions, with reductions observed across all major diagnostic categories, except for respiratory diseases [5]. Within the cardiovascular domain, non-elective diagnostic-related group (DRG) classes for stroke, transient ischemic attack, and heart failure, including cardiogenic shock, saw decreases of 15%, 27%, and 13%, respectively. Concurrently, a 27% decrease in procedures related to circulatory disorders was recorded [5].

The pandemic period witnessed a global decline in hospital admissions for heart failure (HF), ranging from 23.4% to 62% [8]. In Croatia, our study identified an overall 9% decrease, which is notably less than the worldwide average. Similarly, global admissions for ACS patients substantially decreased (40 to 50%) [9], while our data show that the reduction was only slightly higher than 10%, considerably less than in the previously mentioned study.

Focusing specifically on ACS subtypes, a pronounced decrease in STEMI admissions was observed globally (21–56%) [7,8,10], with the Croatian experience showing only a 15% reduction. A systematic review and meta-analysis carried out by Kamarullah et al. showed an even higher global drop in STEMI admissions during the pandemic, reaching up to 80% in some circumstances [11]. Data from Croatia demonstrated a relatively modest decline in STEMI admissions compared to other countries, likely due to the well-established national primary PCI network [12]. A fall in the number of admitted NSTEMI patients was also recorded worldwide, from 33% to 66% [7,8,13]. However, during two pandemic years, the number of admitted NSTEMI patients in Croatia remained similar. Furthermore, admissions for unstable angina (UA) patients during the pandemic were not systematically recorded in the literature. As the least represented part of the ACS continuum, one would reasonably expect the largest drop in admissions for patients presenting with UA. This trend was confirmed in other European countries, such as Western Germany during the government-imposed lockdown period (−23%) [14], and in two single-center studies from Switzerland and India [15,16]. In line with these worldwide trends, we observed a significant decrease in UA admissions in our national dataset, and this decline was the greatest across all ACS subtypes, by the magnitude of 20%. Concomitantly, the utilization of cardiac troponin tests in Croatian hospitals did not decrease but rather increased significantly, suggesting that most patients were likely correctly stratified.

Data on the impact of the COVID-19 pandemic on routine scheduled admissions for coronary chronic syndromes (CCS) are generally lacking, as most studies were focused on emergency departments. Our study clearly showed a sharp drop of 44% in admissions for CCS in Croatia. The reluctance to seek hospital care, as well as a general delay in non-emergency admissions, resulted in more than 2000 fewer case admissions for CCS per year during the pandemic period.

Ultimately, the most crucial quality metric for patients with coronary artery disease (CAD) was the implementation of percutaneous coronary intervention (PCI). According to the systematic reviews and meta-analysis conducted by Kamarullah et al., there was a substantial decline of 72% (from 53% to 97%) in the rate of performed PCIs during the pandemic [11]. In contrast, our data show that the pandemic did not influence the overall number of PCIs performed in Croatia. Interestingly, the number of PCIs conducted in the context of acute myocardial infarction (AMI) even increased in Croatia during the pandemic. This could be attributed to the rejection of the initial literature recommendations favoring thrombolysis by most Croatian cardiologists, and the swift adoption of early recommendations by the Croatian Cardiac Society, emphasizing the use of primary PCI under personal protective equipment [17]. In tandem with the drop in elective admissions for CCS in Croatia, there was a notable 22% decrease in the total number of coronary angiographies performed. Comparatively, an international survey across 108 countries revealed a more extensive global decrease of 55% [18]. While the number of PCIs in the setting of CCS in Croatia showed a reduction of 7.7% during the pandemic, this seems to be much lower than the decrease in overall CCS admissions (44%), suggesting that less stable CCS patients likely still received interventional care during the pandemic period.

The COVID-19 pandemic also revealed a 19.8% reduction in the number of patients undergoing coronary bypass surgery (CABG) in our sample. However, such a decline was less pronounced than in other countries; for example, in Ireland, CABG was performed at 61% of the expected rate in 2020 [19], while in the UK, there was a 51% decline during the lockdown [20]. In Brazil, there was a 25% reduction in CABG procedures in 2020 [21], while data from the USA showed a 35.5% decline in CABG procedures [22].

Anticipated decreases in cadaveric heart registrations and transplants during lockdowns and pandemics were observed globally. A population-based study by Aubert et al., covering 22 countries, reported a decrease in heart transplant rates between 2019 and 2020, ranging from −5.5% to even up to −88.9% worldwide [23]. Across the USA, rates of heart registrations and transplants decreased by 28% and 13%, respectively, during the first two global waves of the COVID-19 pandemic [24]. In Croatia, data from the CHIF database contradict this trend, revealing a higher number of heart transplants performed in Croatia during the pandemic compared to the pre-pandemic period, thus showing an increase of 9.4%. In line with this, during the pandemic period, advanced techniques for heart failure (HF) treatment in Croatia, such as cardiac resynchronization therapy (CRT), saw a significant increase of almost 23%, while the utilization of ventricular assist devices remained virtually unaffected by the pandemic conditions.

It could be anticipated that structural interventional procedures would also experience a decline during pandemics. Global data suggest that transcatheter aortic valve implantation (TAVI) activity decreased by nearly 20% worldwide during the initial months of lockdown compared to the same pre-pandemic period [25]. In contrast, our data show that TAVI procedures in Croatia doubled during the pandemic period. This surge coincided with significant reimbursement efforts by the Croatian Ministry of Health during the early pre-pandemic and pandemic periods. Croatian cardiologists seized the opportunity to enhance their professional endeavors despite the challenges posed by the pandemic, leading to a substantial increase in TAVI utilization. Other advanced interventional procedures, such as transcatheter closure of atrial septal defects, percutaneous transluminal septal myocardial ablation, percutaneous myocardial biopsies, and ventricular assist device implantations, remained consistent in both periods. In contrast, Ireland, for example, experienced a 50% decrease in the latter [21]. As many patients that are severely affected by COVID-19 infection require percutaneous hemodynamic support, it is no surprise that we registered a significant increase in extracorporeal membrane oxygenation (ECMO) procedures during the pandemic period.

At the onset of the pandemic, an increased incidence of bradycardia and relative bradycardia was reported in patients with COVID-19 infection, leading to a rise in pacemaker (PM) implantations [26]. Among COVID-infected patients, it was observed that most devices were implanted due to high-degree or complete atrioventricular block, with a smaller percentage attributed to sick sinus syndrome (SSS) [27]. However, a pan-European observational registry across 15 centers from 12 countries revealed a relative decrease in the percentage of bradycardia/atrioventricular blocks (AVB) in acute cardiac settings from 14% in 2019 to 11% in 2020 [4]. The overall hospital admissions of patients with second/third-degree AVB in Croatia remained similar to the pre-pandemic period, while admissions due to SSS decreased significantly. Considering that AVBs primarily have urgent indications and SSS represents mostly elective indication for pacemaker implantation, it seems that the Croatian healthcare system effectively addressed patients in acute situations related to bradycardic/AVB complications during the pandemic. Although pacemaker implantation procedures (VVI and DDD) decreased by around 15% during the pandemic in Croatia, this decline was more moderate than those reported in other studies. For instance, a survey conducted by the Italian Association of Arrhythmology and Cardiac Pacing revealed that 50% of centers reported a reduction of more than 50% in elective pacemaker implantations [28]. Similar trends were observed in a Spanish study, with a total decrease of 35.2% in the number of preferential/urgent pacemaker implantations [29]. Regional studies further supported these findings, showing significant reductions in pacemaker implantations, including a 28% decrease in the Veneto Region in Italy [30], a 42.3% decrease in overall procedures in Southern Italy [31], a 73% reduction in one Peruvian clinical hospital center [32], and a 54.7% decrease across nine hospitals in Catalonia [33].

Concerning implantable cardioverter–defibrillator (ICD) implantations during the COVID-19 pandemic, some data are available. For instance, in Italy, 92.9% of centers reported a reduction in the number of implantations for primary prevention and 72.6% for secondary prevention [28]. In contrast to this trend, there was a significant increase in the number of ICD implantations in Croatia, with a 15% rise during the pandemic period. We further show data on atrial tachyarrhythmias. It has been previously recognized that atrial fibrillation (AF) is the most prevalent sustained cardiac tachyarrhythmia, prompting medical attention in adults. The current estimated prevalence of AF in adults ranges between 2% and 4% [34], with a lifetime risk of approximately 1 in 3 individuals of European ancestry at the index age of 55 years [35,36]. A pan-European observational registry focusing on acute cardiac settings during COVID-19 pandemic indicated a notable decrease of more than 30% in admission of patients with atrial arrhythmias [32]. Simultaneously, hospital admissions for AF or atrial flutter also experienced a significant reduction in Croatia, albeit to a lesser extent compared to other countries, likely indicating fewer undiagnosed cases and fewer patients with missed anticoagulant medications and subsequent strokes.

Regarding standard cardiovascular imaging procedures, an international survey conducted among 909 centers in 108 countries reported a decrease in cardiovascular procedures by 42% from March 2019 to March 2020 and a further reduction of 64% from March 2019 to April 2020 [20]. Specifically, transthoracic echocardiography (TTE) procedures decreased by 59% in this global assessment. Our data, limited to admitted patients, reveal a 15% decrease in TTE procedures during the pandemic. On the other hand, there was even an increase in transesophageal ultrasound procedures among the Croatian hospital population. Similarly, worldwide computed tomography angiography (CCTA) saw a significant reduction of 54% [20], but this reduction was significantly lower than that observed for exercise stress tests (84%), suggesting a higher utilization of non-stress modalities for coronary artery disease assessment [37]. The majority of CCTA procedures in Croatia occurred in an outpatient setting, and although these specific data are unavailable for our study, among Croatian in-hospital patients, a 15% decrease in CCTA utilization was observed. In the USA, the pandemic saw a 72% decrease in the number of cardiac magnetic resonance imaging (CMR) procedures [19]. Similar trends were observed in certain centers in southern Italy, reflecting a significant reduction in CMR use [38]. Assessing the presence and severity of myocardial injury, CMR emerges as a clinically valuable diagnostic tool, and in contrast to global trends, Croatia experienced an increase regarding the in-hospital use of CMR imaging during the pandemic period. Concurrently, the use of laboratory tests for HF patients, such as natriuretic peptides, more than doubled during the pandemic. Conversely, some routine biochemical tests in CAD patients, such as lipid profiles, significantly decreased in Croatian hospitals.

It is somewhat surprising that in Croatia, the reluctance of patients to seek hospital care due to fear of infection, government lockdowns, and alarming media reports had a less strong impact on cardiovascular service delivery compared to other countries. While initially understandable, the motivation to protect an already overburdened healthcare system likely weakened over time, a trend observed in various societies, including Croatia [39].

This relative success in managing the pandemic compared to other similar countries could be attributed to the Croatian Ministry of Health’s strategy of centralizing COVID-19 patients in newly established regional “COVID-19 hospitals”. This allowed other “non-COVID-19” hospitals to continue functioning normally in the provision of regular medical care of COVID-negative patients. The strategy to allocate all acutely ill COVID-19-positive patients requiring hospitalization to dedicated facilities, coupled with the Croatian healthcare staff’s adaptability honed through experiences like the Homeland War in the 1990s and recent devastating earthquakes, likely enabled Croatian healthcare system to organize more effectively during this crisis compared to routine situations. These are some of the putative explanations from an organizational and health policy perspective that might explain the differences observed in Croatia vs. other countries concerning the cardiovascular healthcare utilization during COVID-19 pandemic. Some societal factors might have contributed to described response during the COVID-19 pandemic such as the fact that many healthcare professionals providing care during COVID-19 pandemic also participated in the Croatian Homeland War and were trained in catastrophic circumstances during wartime. It could also be that the COVID-19 pandemic imposed a much larger number of patient cases requiring cardiovascular procedures and interventions that we describe and this might not necessarily indicate the absence of disruption in the Croatian cardiovascular services system. There, of course, might be other unmeasured circumstances and confounders that affected this response; however, that remains beyond the scope of the current paper and should be addressed by future research.

There are some limitations to this analysis. For example, we did not capture or analyze the potential effects of different pandemic phases on cardiovascular healthcare utilization, as it should be acknowledged that the spread and severity of virus variants and public health measures changed over the observed pandemic period and this might have had an impact on the outcomes that we report. Furthermore, we did not explore patient outcomes such as mortality in our analysis as we did not design our study to capture these events but rather focused on procedural and diagnostic aspects of cardiovascular healthcare. Finally, data obtained from the Croatian Health Insurance Fund do not provide detailed and granular information on several potentially important variables or confounders but offer only big-scale descriptive information on cardiovascular service utilization which has hindered the possibility of executing more complex analyses of current data.

## 5. Conclusions

Our analysis shows that the Croatian cardiovascular healthcare system successfully weathered the COVID-19 pandemic by maintaining or even increasing the number of certain cardiovascular procedures and services. This could be attributed to a combination of factors, including the healthcare system’s structure, proactive strategies, and the experience of its professionals in handling crises. Further research is needed to fully elucidate and identify factors that potentially contributed to such outcomes.

## Figures and Tables

**Table 1:** Incidence rate ratios and utilization of heart failure-related healthcare services during pre-pandemic and pandemic period.

Variable	Pre-Pandemic N per Year (2017 to 2019)	Pandemic N per Year (2020 to 2021)	IRR (95% CI)	*p*-Value
HF admissions	6021	5474	0.909 (0.876–0.943)	<0.0001
CRT implantations	162	200	1.228 (0.993–1.521)	0.0510
VAD implantations	27	25	0.926 (0.515–1.657)	0.784
ECMO uses	234	317	1.355 (1.140–1.611)	0.0004
Heart transplants	32	36	1.094 (0.658–1.825)	0.7162

Abbreviations: CRT—cardiac resynchronization therapy; ECMO—extracorporeal membranous oxygenation; HF—heart failure; VAD—ventricular assist device.

**Table 2:** Incidence rate ratios and number of ACS- and CCS-related hospitalizations during pre-pandemic and pandemic period.

Variable	Pre-Pandemic N per Year (2017 to 2019)	Pandemic N per Year (2020 to 2021)	IRR (95% CI)	*p*-Value
ACS, overall	11,481	10,275	0.895 (0.871–0.919)	<0.0001
STEMI	4311	3658	0.845 (0.808–0.883)	<0.0001
NSTEMI	5261	5107	0.971 (0.934–1.009)	0.1303
Unstable angina	1910	1525	0.798 (0.746–0.854)	<0.0001
CCS	6270	4122	0.657 (0.632–0.684)	<0.0001

Abbreviations: ACS—acute coronary syndrome; CCS—chronic coronary syndrome; STEMI—ST elevation myocardial infarction; NSTEMI—non-ST-segment elevation myocardial infarction.

**Table 3:** Incidence rate ratios and number of interventional cardiology procedures and CABG and advanced interventional procedures performed during the pre-pandemic and COVID-19 pandemic period.

Variable	Pre-Pandemic N per Year (2017 to 2019)	Pandemic N per Year (2020 to 2021)	IRR (95% CI)	*p*-Value
CAG and CC	25,938	20,134	0.776 (0.762–0.791)	<0.0001
PCI, total	8633	8505	0.985 (0.956–1.015)	0.3402
PCI in ACS	4214	4424	1.050 (1.006–1.095)	0.0238
PCI in CCS	4419	4081	0.923 (0.885–0.964)	0.0002
CAG in ACS without PCI	1266	1058	0.836 (0.769–0.907)	<0.0001
CABG	1113	894	0.802 (0.734–0.877)	<0.0001
TAVI	91	183	2.010 (1.556–2.615)	<0.0001
ASD closure, transcatheter	61	47	0.770 (0.515–1.146)	0.1796
PTSMA	10	3	0.300 (0.0530–1.165)	0.0574
Endomyocardial biopsy	264	244	0.924 (0.773–1.104)	0.3753

Abbreviations: ACS—acute coronary syndrome; ASD—atrial septal defect; CAG and CC—coronary angiography and cardiac catheterizations; CABG—coronary artery bypass grafting; CCS—chronic coronary syndrome; PCI—percutaneous coronary intervention; PTSMA—percutaneous transluminal septal myocardial ablation; TAVI—transcatheter aortic valve implantation.

**Table 4:** Incidence rate ratios of utilization of cardiovascular imaging modalities and common laboratory tests during the pre-pandemic and COVID-19 pandemic periods.

Variable	Pre-Pandemic N per Year (2017 to 2019)	Pandemic N per Year (2020 to 2021)	IRR (95% CI)	*p*-Value
TTE	55,003	46,880	0.852 (0.842–0.863)	<0.0001
TEE	3981	4357	1.094 (1.048–1.143)	<0.0001
CTCA/calcium scoring	374	318	0.850 (0.730–0.990)	0.0333
Cardiac radionuclide imaging	133	105	0.797 (0.612–1.036)	0.0811
Cardiac MRI	283	369	1.304 (1.114–1.528)	0.0008
Lipid profile	260,392	217,043	0.833 (0.829–0.838)	<0.0001
Troponin testing	103,504	108,181	1.045 (1.036–1.054)	<0.0001
Natriuretic peptide testing	19,830	42,787	2.158 (2.122–2.194)	<0.0001

Abbreviations: CTCA—computerized tomography coronary angiography; MRI—magnetic resonance imaging; TEE—transesophageal echocardiography; TTE—transthoracic echocardiography.

**Table 5:** The number of admitted patients due to bradycardia/heart atrioventricular blocks, atrial fibrillation/flutter, and utilization of pacemaker and ICD implantations during the pre-pandemic and pandemic periods.

Variable	Pre-Pandemic N per Year (2017 to 2019)	Pandemic N per Year (2020 to 2021)	IRR (95% CI)	*p*-Value
AV block (2nd/3rd°) admissions	1567	1489	0.950 (0.885–1.021)	0.1582
SSS admissions	448	356	0.795 (0.689–0.915)	0.0012
Pacemaker implantation	3467	2936	0.847 (0.806–0.890	<0.0001
ICD implantations	608	699	1.150 (1.030–1.284)	0.0118
Atrial fibrillation/flutter admissions	4839	4029	0.833 (0.798–0.868)	<0.0001

Abbreviations: AV—atrioventricular; ICD—implantable cardioverter defibrillator; SSS—sick sinus syndrome.

## Data Availability

Data are contained within the article.
